# Genetics of hearing loss in the Arab population of Northern Israel

**DOI:** 10.1038/s41431-018-0218-z

**Published:** 2018-08-23

**Authors:** Nada Danial-Farran, Zippora Brownstein, Suleyman Gulsuner, Luna Tammer, Morad Khayat, Ola Aleme, Elena Chervinsky, Olfat Aboleile Zoubi, Tom Walsh, Gil Ast, Mary-Claire King, Karen B. Avraham, Stavit A. Shalev

**Affiliations:** 1 Genetics Institute, Emek Medical Center, Afula, Israel; 2 Rappaport Faculty of Medicine, Technion-Israel Institute of Technology, Haifa, Israel; 3 Department of Human Molecular Genetics and Biochemistry, Sackler Faculty of Medicine and Sagol School of Neuroscience, Tel Aviv University, Tel Aviv, Israel; 4 Department of Medicine, Division of Medical Genetics, University of Washington, Seattle, WA USA

## Abstract

For multiple generations, much of the Arab population of Northern Israel has lived in communities with consanguineous marriages and large families. These communities have been particularly cooperative and informative for understanding the genetics of recessive traits. We studied the genetics of hearing loss in this population, evaluating 168 families from 46 different villages. All families were screened for founder variants by Sanger sequencing and 13 families were further evaluated by sequencing all known genes for hearing loss using our targeted gene panel HEar-Seq. Deafness in 34 of 168 families (20%) was explained by founder variants in *GJB2*, *SLC26A4*, or *OTOF*. In 6 of 13 families (46%) evaluated using HEar-Seq, deafness was explained by damaging alleles of *SLC26A4, MYO15A, OTOG, LOXHD1*, and *TBC1D24*. In some genes critical to hearing, it is particularly difficult to interpret variants that might affect splicing, because the genes are not expressed in accessible tissue. To address this problem for possible splice-altering variants of *MYO15A*, we evaluated minigenes transfected into HEK293 cells. Results revealed exon skipping in the message of *MYO15A* c.9083+6T>A, and intron retention in the message of *MYO15A* c.8340G>A, in each case leading to a premature stop and consistent with co-segregation of homozygosity for each variant with hearing loss. The profile of genetics of hearing loss in this population reflects the genetic heterogeneity of hearing loss and the usefulness of synthetic technologies to evaluate potentially causal variants in genes not expressed in accessible tissues.

## Introduction

**Fig. 1:**
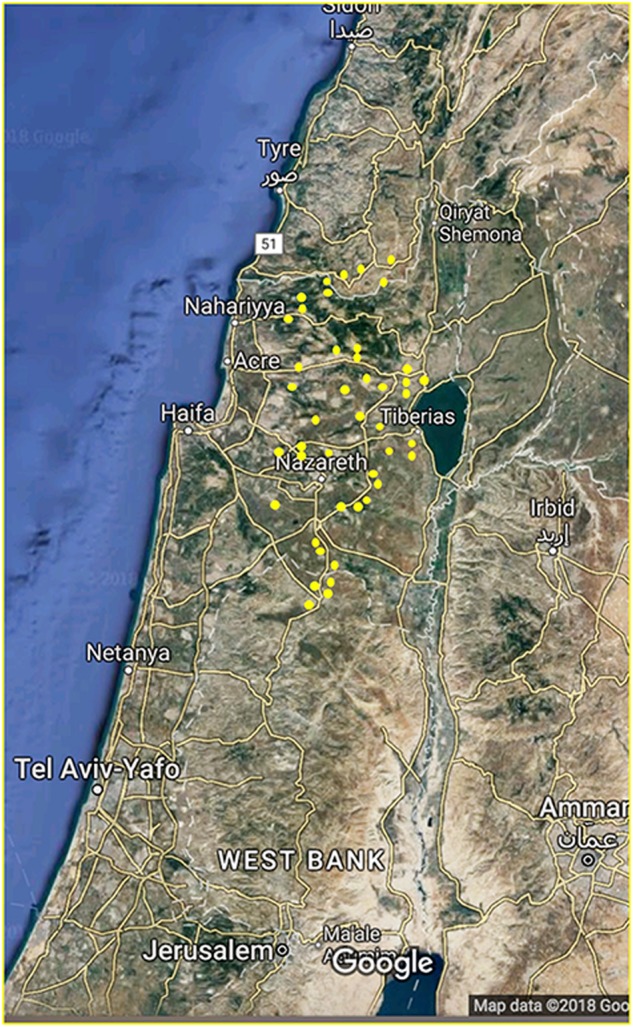
Northern Israel, with home villages of the participants shown in yellow. Reproduced with permission from Google, Mapa GISrael, ORION-ME. (https://www.google.com/maps/@32.6499621,35.4502344,224900m/data=!3m1!1e3?hl=en)

The identification of alleles causing deafness has advanced dramatically in the past decade as a result of the availability of technologies such as next-generation sequencing (NGS) [1, 2]. Applying the NGS technology to families with high consanguinity, living in isolated populations, holds promising potential for characterizing rare alleles causing recessive phenotypes, such as hereditary hearing loss. We focused on the Arab population from northern Israel, the majority of whom live in villages that were founded by a few individuals roughly ten generations ago [3] (Fig. 1). These communities are relatively isolated due to the preference for consanguineous marriages. Occasionally, a rare variant may expand among the descendants of the carriers through a founder effect mechanism and become prevalent in specific villages. Deciphering the dispersion of variants in these communities [4] facilitates relevant and precise genetic counseling for the families. Moreover, acknowledging the effect of genetic variation on a hearing disability might be the cornerstone of future research directed toward restoring hearing.

Our cohort was comprised of 168 Arab families with non-syndromic hearing loss (NSHL). The members of the population live in 46 different villages in the north of Israel, with long traditions of consanguineous marriage and a long history of endogamy [5]. In this study, we used NGS technology, which led to the identification of novel alleles in *SLC26A4, MYO15A, OTOG, LOXHD1*, and *TBC1D24* that are associated with deafness. In addition, we also examined the consequences of two splicing variants in the *MYO15A* gene that we identified in these families.

## Subjects and methods

### Families

This study was approved by the Ethical Committees of the Emek Medical Center and Tel Aviv University, the Helsinki Committee of the Israel Ministry of Health, and by the Human Subjects Division of the University of Washington. A medical history was obtained from all participating individuals, including a detailed pedigree emphasizing hearing loss and other relevant symptoms. Of the 168 families, 151 reported consanguinity. Affected individuals were asked for details about the severity of hearing loss, age of onset, symmetry of hearing loss, use of hearing aids or cochlear implants, experience of tinnitus, exposure to ototoxic drugs and/or noise, pathological conditions of the inner ear, and vestibular function. After acquiring signed informed consent, we obtained fresh blood samples for DNA extraction.

###  DNA sequencing

DNA extraction was performed with the DNeasy Blood & Tissue Kit (Qiagen, Hilden, Germany). First, DNA from all families was sequenced for *GJB2* by Sanger sequencing, using the BigDye Terminator Cycle Sequencing Kit (Applied Biosystems, Foster City, CA, USA), and analyzed using the ABI PRISM 3100 Genetic Analyzer (Applied Biosystems) according to the manufacturer’s instructions. We then applied massively parallel sequencing (MPS) of deafness genes using the HEar-Seq4 panel, modified from Hear-Seq2 [6]. Briefly, 375 protein-coding genes and microRNAs (miRNAs) were captured using the Agilent SureSelect Target Enrichment System (Agilent, Santa Clara, CA, USA). The list of genes and miRNAs appears in Supplementary Table S1, indicating source of gene, mode of inheritance and association to syndromes. The UCSC Genome Browser hg19 coordinates of the genes were submitted to eArray (Agilent Technologies) in order to design cRNA oligonucleotides to cover all exons and 10 bp of flanking introns. The choice of genes was based on their association with deafness in humans and mice or literature evidence for their expression in the cochlea (Supplementary Table S1). Molecular barcodes were assigned, and 48 samples were multiplexed and sequenced in a single flow-cell of the Illumina HiSeq with 100 bp paired-end reads. MPS was performed at the Technion High-Throughput Sequencing Center (Haifa, Israel) and the King laboratory. Results were validated by Sanger sequencing and checked for co-segregation with hearing loss in the families. The prevalence of allele frequencies was examined in the hearing-impaired population and compared to that in the relevant ethnic population. Variants were uploaded to the LOVD locus-specific database (www.lovd.nl/) and ClinVar (https://www.ncbi.nlm.nih.gov/clinvar/).

### In silico validation of splice variants

Six prediction tools were used to estimate the splice site strength of different sequences. Five of these, SpliceSiteFinder, GeneSplicer, Splice Site Prediction by Neural Network, MaxEntScan, and Human Splicing Finder are available through Alamut software v.2.3 (http://www.interactive-biosoftware.com). The sixth is the Analyzer Splice Tool (http://host-ibis2.tau.ac.il/ssat/SpliceSiteFrame.htm).

### Splicing assays

Two pairs of minigene clones were designed for the *MYO15A* variants: c.8340G>A, and c.9083+6T>A. Each pair included one plasmid with the wild-type sequence, and a second plasmid with the mutant sequence. For c.8340G>A, the insert was amplified from exon 45 downstream to exon 47 (chr17:g.18,154,705–18,155,383), and for c.9083+6T>A from exon 51 downstream to exon 53 (chr17:g.18,157,808–18,158,983). PCR amplification from DNA of wild-type and homozygote family members was carried out using the primers: 5′-GCTGCCAACGGAGCCAGG-3′ and 5′-CAGAACCTTGGAGGACATGC-3′; 5′-CCTGCCTTCGCATCTCTG-3′ and 5′-TGACCATCCTCAGGAGTTTGA-3′; respectively. A PCR reaction was designed to open the pcDNA™5/FRT plasmid using KAPA HiFi HotStart ReadyMixPCR Kit (Kapa Biosystems, Wilmington, DE, USA), with the primers: pcDNA5-FRT-Fw: GGCGGCCGCTCGAGTCTA, and pcDNA5-FRT-Rv: CGCTAGAGTCCGGAGGCT, length: 5062 bp.

Flp-In™−293 cell lines (Thermo Fisher Scientific, Waltham, MA, USA) were cultured in Dulbecco's Modified Eagle’s Medium (DMEM) supplemented with 4.5 g/ml glucose (Biological Industries, Beit Haemek, Israel), 10% Fetal Calf Serum (FCS), 0.29 mg/ml l-glutamine, 100 U/ml penicillin, 0.1 mg/ml streptomycin, and 1 U/ml nystatin (Biological Industries). Cells were grown in 100 mm culture dishes under standard conditions at 37 °C in a humidified atmosphere with 5% CO_2_.

For the minigene assay, ~250 K cells were seeded in 100 mm culture dishes. Twenty-four hours later, the cells were transfected using TransIT®-LT1 transfection reagent (Mirus Bio, Madison, WI, USA), according to the manufacturer’s protocol. After 48 h, RNA was extracted from the cells.

For *MYO15A* c.8340G>A, stable cell lines were developed by co-transfection of ~250 K Flp-In™−293 cells seeded in 100 mm culture dishes, with the pcDNA™5/FRT/TO expression construct and the pOG44 Flp-Recombinase Expression Vector, in a ratio of 1:9. Forty-eight hours later, the cells were harvested and transferred to 15 cm culture dishes. Stable transfectants were grown for three weeks and selected with Hygromycin B antibiotics.

### RNA isolation and RT-PCR

Total RNA extraction was performed using TRI Reagent (Merck, Darmstadt, Germany). For RT-PCR reactions, 2 µg of total RNA was amplified by reverse transcriptase (RT) in a final volume of 20 µl containing: 5 mM DTT, 0.5 mM dNTPs, 2.5 µM oligo d(T) primer, 2 U of SuperScript® III Reverse Transcriptase (Thermo Fisher Scientific), and RT buffer. The final mixture was incubated for 1 h at 50 °C, and then 5 min at 85 °C to inactivate the enzymes.

To specifically amplify the spliced cDNA products derived from the expressed minigene, rather than the endogenous gene, we designed a pair of primers: a forward pcDNA™5/FRT/TO-specific primer and a reverse primer specific to the inserted minigene 3′ sequence. Amplification was performed for 30 cycles, 94 °C for 30 s, 60 °C for 45 s and 72 °C for 1 min. The products were confirmed by Sanger sequencing.

## Results

### Sanger sequencing screen for known variants

Affected probands were screened by Sanger sequencing, for the coding sequence in *GJB2*, and for one prevalent variant in *SLC26A4*. This screen resolved 34 cases out of 168. The variants detected in *GJB2* (NM_004004.5) were: c.35delG (18/34), c.229T>C (3/34), c.−23+1G>A (2/34), c.301_303delGAG (1/34), and c.167delT (1/34), all in the homozygous state. Two additional compound heterozygotes were detected by the initial screening, *GJB2* c.[35delG];[109G>A] and c.[35delG];[298C>T] (2/34). Moreover, we screened for the *SLC26A4* c.1197delT variant, as it was previously found in the northern Israel Arab population [7] and identified seven deaf individuals who were homozygous for this variant. Overall, the initial screen yielded a 20.2% (34/168) detection rate.

### Targeted deafness gene panel (MPS)

**Fig. 2:**
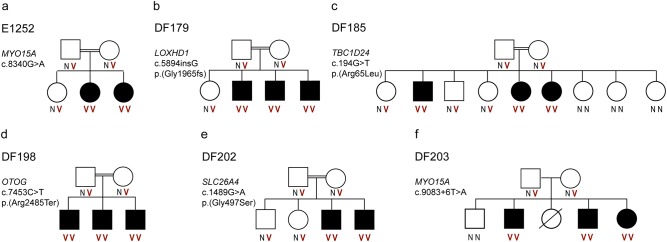
Pedigrees of the families with NSHL investigated by the HEar-Seq deafness panel and the segregation analysis. **a** Family E1252. **b** Family DF179. **c** Family DF185. **d** Family DF198. **e** Family DF202. **f** Family DF203. N, wild type; V, variant

Thirteen unsolved families with congenital NSHL were analyzed by high-throughput sequencing with our targeted deafness panel, HEar-Seq (Supplementary Table S1) [6]. All families presented a recessive mode of inheritance with non-syndromic, moderate to severe or profound hearing loss, with congenital or prelingual onset (Table 1). The analysis of NGS data led to the identification of five novel variants in the known deafness genes *MYO15A*, *LOXHD1*, *TBC1D24*, and *OTOG*, all of which were associated with pathogenicity, and categorized according to the ACMG guidelines [4] (Table 1). HGVS nomenclature and LOVD IDs are presented in Supplementary Table S2. An additional known disease-causing variant was identified in the *SLC26A4* gene (Table 1). All variants segregated with hearing loss in the families (Fig. 2). As part of the validation interpretation, we screened for all the identified variants in a population comprised of hearing controls, sharing the same ethnicity. None of the identified variants were present in any of the chromosome samples. Subsequently, we sequenced the identified variants across 120 undiagnosed hearing impaired probands. A diagnosis for one family revealed that two deaf siblings from the same village as the DF198 family are homozygous for *OTOG* c.7453C>T, p.(Arg2485Ter).

**Table 1:** Variants identified in families with non-syndromic hearing loss by HEar-Seq

Family	HL phenotype, onset	Gene	Genomic coordinate (hg38), rs number (dbSNP)	cDNA position (RefSeq mRNA accession)	Effect	Allele frequency in hearing controls (chromosomes)	Allele frequency in unrelated deaf (chromosomes)	ACMG classification [4]
E1252	Profound, congenital	*MYO15A*	chr17:18,155,225, rs878853228	c.8340G>A (NM_016239.3)	Intron 46 retention	0 (220)	0 (194)	Pathogenic PS3
DF179	Profound, prelingual	*LOXHD1*	chr18:46,485,121, rs878853231	c.5894dupG (NM_144612.6)	p.(Gly1965fs)	0 (264)	0 (194)	Pathogenic PVS1
DF185	Profound, congenital	*TBC1D24*	chr16:2,496,342, rs878853232	c.194G>T (NM_001199107.1)	p.(Arg65Leu)	0 (206)	0 (194)	Pathogenic PP1
DF198	Moderate-severe, prelingual	*OTOG*	chr11:17,634,218, rs866476223	c.7453C>T (NM_001277269.1)	p.(Arg2485Ter)	0 (220)	0.005 (194)	Pathogenic PVS1
DF202	Profound, congenital	*SLC26A4*	chr7:107,695,984, rs111033308	c.1489G>A (NM_000441.1)	p.(Gly497Ser)		0 (194)	
DF203	Profound, congenital	*MYO15A*	chr17:18,158,644, none	c.9083+6T>A (NM_016239.3)	Skipping exon 52	0 (220)	0 (194)	Pathogenic PS3

### Validation of splicing variants

**Fig. 3:**
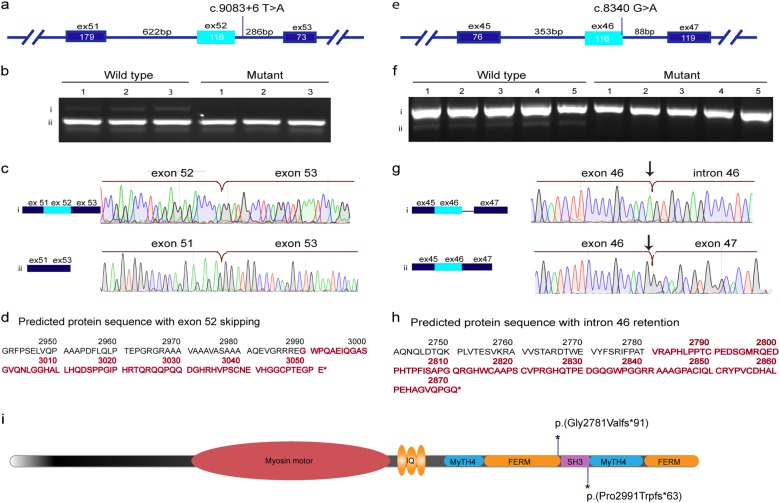
Analysis of the expression pattern of the wild-type and mutant alleles of *MYO15A*. **a**, **e** A schematic representation of the constructs designed for minigene (for c.9083+6T>A) and stable cell line (for c.8340G>A) experiments, respectively. For each experiment two plasmids were prepared, one with the wild-type sequence and second with the investigated variant. **b**, **f** Agarose gel electrophoresis of cDNA PCR products prepared from RNA extracted from transfected cell lines. **c**, **g** A sequence chromatogram of each isoform detected by gel electrophoresis. **d**, **h** The predicted amino acid sequence of the protein that is truncated as a result of alternative splicing. **i** Schematic representation of the myosin XVA protein [22] shows the location of each predicted truncation due to the splice variants

We further investigated the effects of two variants in *MYO15A*: c.9083+6T>A, exon 52, and c.8340G>A, p.(Thr2780Thr), in the last nucleotide of exon 46, as we hypothesized that they may affect splicing. Because the tissues that express this gene are inaccessible, we could not obtain RNA from the patients in order to evaluate the effect of the variants on expression directly. For c.9083+6T>A, a transient minigene experiment revealed a differential expression pattern between the wild type and mutated plasmid. This was then examined by RT-PCR after extraction of RNA. The results revealed that there are two isoforms that are expressed normally: one with full exon inclusion, and the second without exon 52 (Fig. 3a). In contrast, the mutant sequence expressed only the second isoform without exon 52.

The minigene assay failed to express the insert with c.8430G>A using a transient vector. For this reason, we used the Flp-In™-System to generate two stably transfected 293 cell lines, one with the wild type and the second with the mutant. It is important to note that the two minigenes were inserted into the same genomic locus and in the same orientation. The splicing pattern was then examined by RT-PCR after extraction of RNA. Genomic DNA was extracted from each cell line in order to confirm that the integrated minigene did not acquire any unforeseen variant. In the wild-type stable cell line, the RT-PCR results included two mRNA products: the full exon inclusion isoform, and a second isoform with retention of intron 46. In the mutated cell line, the full exon inclusion isoform was absent in all examined colonies (*n* = 5), and only the isoform with intron retention was detected (*n* = 5) (Fig. 3e–g). Importantly, the G>A variant in the last nucleotide of exon 46 eliminates the full exon inclusion isoform, indicating that this variant impairs splicing of exon 46.

## Discussion

The objective of the current study was to identify genetic causes of hearing impairment in consanguineous families living in northern Israel. The detection rate of alleles affecting function in our cohort by high-throughput sequencing was 47% (6/13), and included novel alleles associated with hearing loss. This outcome is in agreement with other studies conducted in various endogamous as opposed to non-consanguineous populations worldwide [8–10]. Our study highlights the advantages of studying isolated populations for detecting rare recessive variants associated with hearing impairment. Most of the Arab population in Israel live in villages and towns that were founded by few founders about ten generations ago and is characterized by frequent consanguineous/endogamous marriages and a high fertility rate [11]. Each of the localities may therefore be regarded as a small isolate. According to their geographic distribution, the genetic diseases may be classified into diseases that are relatively frequent in all the population, such as thalassemia, usually caused by ancient variants. Diseases that are frequent in more than one community, and those which are confined to a local community, were found to be caused by variants that are either relatively recent or were introduced recently in the population, most of them in a homozygous state. Therefore, it is probable that most of the genetic diseases in this population is due to founder effect.

All the variants detected in our cohort are recessive and the hearing-impaired individuals represent a homozygous state. The novel variant, c.5894dupG in the *LOXHD1* gene, is a frameshift variant that is predicted to damage the sequence downstream of position 1695, a genomic location that is evolutionarily conserved. This variant was found in three affected siblings in a homozygous state and was absent in our hearing and hearing-impaired cohorts. The variant in family DF198, c.7453C>T, in the *OTOG* gene, is a nonsense variant. Three affected siblings had this variant in a homozygous state. Subsequently, one deaf individual from our hearing-impairment cohort was found to be a homozygote, and then, his deaf sibling was also tested and found to be homozygous for this variant. Of note, these two individuals excluded familial relations with family DF198, although they do live in the same village. Hence, although the haplotypes were not investigated, we assume that they are from a common founder.

The variant c.194G>T, p.(Arg65Leu) in *TBC1D24* that was detected in family DF185, was found in three homozygous siblings, and segregation was supported by testing additional eight hearing family members. We did not find this variant in either normal or affected cohorts matched for the same ethnic background. A variant located five amino acids downstream, p.(Asp70Tyr), was previously detected in a consanguineous family with profound NSHL, as in family DF185 [12]. Of note, some variants in *TBC1D24* are associated with deafness, onychodystrophy, osteodystrophy, mental retardation and seizures (DOORS). The affected members of family DF185 did not exhibit any symptoms other than deafness, as determined by detailed medical examinations. Superimposing the human variants on the crystal structure of the *Drosophila* ortholog defined the putative function of some of these residues, based on their location or binding with PI(4,5)P2 [13].

The additional novel variants in *MYO15A* were detected in two families; and neither were present in our hearing or affected cohorts. Further investigation revealed that these variants are located in critical splicing regions, with strong evidence for associated pathogenicity, according to the location and to in silico analysis (Table 2). Splicing variants might alter normal splicing and result in non-functional gene products; however, different splicing patterns might actually produce different products that have a benign or even favorable effect [14]. Previous studies have shown that exonic and intronic variants that were considered to be variants with unknown significance were subsequently implicated in diseases by studying their effect on splicing [15]. Therefore, splicing analysis on the RNA level is a relevant starting point to determine the potential pathogenicity of a variant. Unfortunately, the evaluation of splicing variants can be limited by the lack of RNA samples, as is the case for inner ear-specific genes that are not expressed in accessible tissues. In such cases, our minigene experiments with transient vectors are an attractive alternative that can be used to investigate the effect of splicing variants on translation. Studies have shown 100% concordance when comparing the effect of splicing variants on RNA and minigene constructs [16, 17]. This approach has been used to study other variants implicated in hearing loss, including for *MYO15A*, *STRC*, *TJP2* and *USH2A* [18, 19], The minigene experiment described here was designed to evaluate the expression pattern of c.9083+6T>A and revealed a differential splicing pattern between the wild type and the mutant alleles. Expression of the wild-type sequence was detected in the cell lines transfected with the wild-type minigene plasmid, but not with the mutated sequence. This was confirmed by sequencing. The minigene results provide evidence of the aberrant splicing pattern, which may be caused by disruption of U1 snRNP binding to the 5′ splice site that prevents splicing initiation [13] and leads to skipping exon 52.

**Table 2:** Prediction tools to evaluate the donor splicing site sequence strength

	Splice site finder (0–100)	Max Ent scan (0–12)	NNSPLICE (0–1)	Gene splicer (0–15)	Human splice finder (0–100)	Analyzer splice tool (0–100)
*MYO15A*, c.8340 (G) Ref.	70.03	7.2	0.61	9.39	82	69.54
*MYO15A*, c.8340 (A) Alt.	NI	0.23	NI	5.19	71.42	57.63
Decrease in splicing strength (%)	–	58%	–	28%	10.58%	11.91%
*MYO15A*, c.9083+6 (T) Ref.	74.59	8.3	0.9	9.3	83.5	73.91
*MYO15A*, c.9083 (A) Atl.	NI	3.7	NI	6	81.5	68.64
Decrease in splicing strength (%)	–	38.3%	–	22%	2%	5.27%

*NI* not identified, *WT* Wild type

It is important to appreciate that minigene experiments may not always be an appropriate approach to studying splicing variants. This was illustrated by the case of family E1252 (Table 1), where we investigated the consequences of the novel splicing variant c.8340G>A in the *MYO15A* gene, located in the last base pair of exon 46. In this case, the minigene technique failed to resolve the expression pattern of this genomic region, possibly because of the high GC content, and short length (88 bp) of the intron. Further analysis, using a stable cell line, successfully confirmed the altered splicing pattern. Expression of an isoform with intron retention was seen in the mutated cells but there was no full exon inclusion isoform with exon 46. The results were confirmed by sequencing.

We can conclude from the expression patterns of both c.8340G>A and c.9083+6T>A that the altered sequences result in aberrant proteins and that this is responsible for the lack of normal mRNA isoforms in both tested families. We predict that a defective reading frame results in a truncated non-functional protein lacking the tail that contains three critical domains: SH3, MyTH4, and FERM, a region that is crucial for normal hearing (Fig. 3d, h, i) [20, 21].

In summary, diagnosing the genetic basis of deafness is a first crucial step in understanding the underlying pathological mechanisms causing hearing impairment, and might contribute to our knowledge of the physiological process of hearing. From a clinical perspective, our findings have dramatically improved the clinical management of patients and can be used to guide the genetic counseling provided to affected individuals and their family members. Finally, investigating the consequences of a novel variant may be the cornerstone to developing future gene-specific therapies.

## Electronic supplementary material

Supplementary Table S1

Supplementary Table S2
