# Supplementary material for: Genetics of hearing loss in the Arab population of Northern Israel
Source: Eur J Hum Genet. 2018 Aug 23;26(12):1840–7. doi: 10.1038/s41431-018-0218-z (PMC6244407; doi:10.1038/s41431-018-0218-z)
Supplement: Supplementary file 2 — Supplementary Table S2 [file 41431_2018_218_MOESM2_ESM.pdf]

Genetics of hearing loss in the Arab population of northern Israel

Nada Danial-Farran, Zippora Brownstein, Suleyman Gulsuner, Luna Tammer, Morad Khayat, Ola Aleme,
Elena Chervinsky, Olfat Aboleile Zoubi, Tom Walsh, Gil Ast, Mary-Claire King, Karen B. Avraham, Stavit A. Shalev

Supplementary Table S2 Complete HGVS nomenclature and LOVD IDs

Family	*LOVD Individual_ID*	*Gene*	*HGVS nomenclature*
E1252	00163627<br>https://databases.lovd.nl/shared/individuals/00163627	MYO15A	NG_011634.1(NM_016239.3):c.8340G>A, exon 46
DF179	00163776<br>https://databases.lovd.nl/shared/individuals/00163776	*LOXHD1*	NG_016646.1(NM_144612.6):c.5894dupG, exon 38
DF185	0163777<br>https://databases.lovd.nl/shared/individuals/00163777	*TBC1D24*	NG_028170.1(NM_001199107[p.1):c.194G>T, exon 2
DF198	00154485<br>https://databases.lovd.nl/shared/individuals/00154485	*OTOG*	NG_033191.1(NM_001277269.1):c.7453C>T, exon 43
DF202	00163814<br>https://databases.lovd.nl/shared/individuals/00163814	*SLC26A4*	NG_008489.1(NM_000441.1):c.1489G>A, exon13
DF203	00154503<br>https://databases.lovd.nl/shared/individuals/00154503	*MYO15A*	NG_011634.1(NM_016239.3):c.9083+6T>A, exon 52
